# Unresectable Primary Enteric‐Type Thymic Adenocarcinoma Treated With FOLFOX Chemotherapy: A Case Report

**DOI:** 10.1002/cnr2.70318

**Published:** 2025-09-04

**Authors:** Carl He, Georgia Bentick, Patrick Hosking, Andrew Mant

**Affiliations:** ^1^ Department of Oncology Eastern Health Melbourne Australia; ^2^ Peter MacCallum Cancer Centre Melbourne Australia; ^3^ Department of Pathology Eastern Health Melbourne Australia

**Keywords:** enteric‐type thymic adenocarcinoma, thymic adenocarcinoma, thymic carcinoma

## Abstract

**Background:**

Enteric‐type thymic adenocarcinomas are an extremely rare and distinct subtype of thymic malignancies, as classified by the 2021 World Health Organization classification of thymic tumors. These tumors exhibit close molecular and morphologic similarity to primary gastrointestinal malignancies. To date, there are no tailored treatment guidelines for enteric‐type thymic adenocarcinoma.

**Case:**

A 65‐year‐old woman was admitted to the Oncology unit of a Melbourne Metropolitan Hospital after presenting with progressive symptoms of thoracic outlet syndrome, where a CT scan identified a localized anterior mediastinal mass measuring up to 7.5 × 6.0 × 6.0 cm (transverse × Anterior–Posterior × Superior–inferior). The mass was deemed unresectable. A core biopsy diagnosed the patient with a primary enteric‐type thymic adenocarcinoma, based on positive immunohistochemical staining for the intestinal markers CK20 and CDX2, positive staining for CD5—a marker commonly associated with thymic carcinoma, and positive staining for CK7—a marker not classically expressed in colonic adenocarcinoma. The patient received five fractions of 20 Gray palliative radiotherapy for superior vena cava syndrome, followed by four cycles of Oxaliplatin/5‐fluorouracil/leucovorin (FOLFOX) chemotherapy. FOLFOX, a common systemic therapy for gastrointestinal adenocarcinomas, was chosen due to the tumor's enteric profile and was selected over the then‐NCCN‐recommended carboplatin/paclitaxel regimen, which did not have trial‐backed evidence of efficacy in thymic adenocarcinomas, particularly adenocarcinomas of the enteric type. After starting on frontline systemic therapy with FOLFOX, restaging scans after Cycle 3 showed a reduction in the size of her anterior mediastinal mass, which further decreased in size after cycle 4. Treatment was then discontinued per the patient's goals of care; however, follow‐up imaging showed ongoing disease stability for 6 months. The patient died of disease 8 months after treatment discontinuation and 13 months after her initial diagnosis.

**Conclusion:**

We report the first case of primary enteric‐type thymic adenocarcinoma treated at an Australian institution, and the first published case using FOLFOX as frontline systemic therapy in a patient with unresectable primary enteric‐type thymic adenocarcinoma. Noting the close molecular resemblance between enteric‐type thymic adenocarcinoma and gastrointestinal malignancies, chemotherapy regimens commonly used in the treatment of gastrointestinal cancer may offer a more effective option than standard frontline therapies used in thymic carcinomas for the management of enteric‐type thymic adenocarcinoma.

## 1 Introduction

Thymic adenocarcinomas are rare subtypes of primary thymic carcinomas [1]. The first documented case was reported by Moriyama et al. in 1989; [2] however, thymic adenocarcinoma was not formally recognized as a histological subtype of thymic carcinoma until 1997 [3]. Thymic adenocarcinomas can be further classified into adenocarcinoma not otherwise specified, low‐grade papillary adenocarcinoma, thymic carcinoma with adenoid cystic carcinoma‐like features, and enteric‐type adenocarcinomas as defined by the WHO 2021 criteria [4]. Enteric‐type thymic adenocarcinomas were first proposed by Moser et al. in 2015 as an individual subtype of thymic adenocarcinoma [5]. However, the first reported case matching the molecular criteria of enteric‐type thymic adenocarcinoma was by Choi et al. in 2003 [6]. Enteric‐type thymic adenocarcinomas are mucinous or nonmucinous adenocarcinomas characterized by the expression of at least one of the intestinal markers: CK20, CDX2, or MUC2 [4]. They are incredibly rare, with 49 total cases reported in the literature to date.

Due to its rarity, no specific treatment guidelines are provided for the management of enteric‐type thymic adenocarcinomas. The NCCN guidelines advise carboplatin/paclitaxel+ramucirumab as the first‐line systemic therapy of choice for all unresectable or metastatic thymic carcinomas, with ramucirumab recently being added to the guidelines in 2025 [7]. The carboplatin/paclitaxel combination has previously demonstrated an overall response rate (ORR) of 21.7%–40% in the treatment of advanced thymic carcinoma [8, 9, 10, 11, 12]. Two prospective phase II trials have evaluated the efficacy of carboplatin/paclitaxel in thymic carcinoma [13]. Lemma et al. evaluated 23 patients with unresectable, invasive, recurrent, or metastatic thymic carcinoma treated with carboplatin/paclitaxel, yielding an ORR of 21.7% and a median progression‐free survival (PFS) of 5.0 months [10]. Hirai assessed this regimen in 39 chemotherapy‐naïve patients with advanced or metastatic thymic carcinoma, demonstrating an ORR of 36% and a median PFS of 7.5 months [11]. The cohort studied by Hirai et al. consisted of 53.8% of patients with squamous cell carcinoma—the most prevalent histologic subtype of thymic carcinoma, [14] but included no confirmed cases of thymic adenocarcinoma. Lemma et al. did not specify the histological subtypes classified within the population of patients with thymic carcinoma studied. Consequently, it is unclear whether the efficacy of this regimen can be generalized to thymic adenocarcinoma, and a future trial specifically investigating systemic treatment for thymic adenocarcinoma may identify a regimen superior to the platinum/taxane combination. A parallel may be drawn from the systemic treatment of non‐small cell lung cancer (NSCLC). Previously, platinum‐based chemotherapy combinations were the mainstay of systemic treatment for advanced NSCLC [15, 16]. A later retrospective analysis of a phase III trial comparing pemetrexed and docetaxel as second‐line chemotherapy in NSCLC identified histology‐specific differences in response: patients with the squamous subtype exhibited superior overall survival (OS) with docetaxel, while those with non‐squamous histology—including adenocarcinoma, large cell carcinoma, and indeterminate subtypes—collectively demonstrated an improved OS with pemetrexed [17]. As a result, frontline chemotherapy for NSCLC is now stratified by histologic subtype, with frontline treatment for squamous NSCLC utilizing paclitaxel plus a platinum agent, and non‐squamous subtypes—including adenocarcinoma—utilizing pemetrexed plus a platinum agent [18].

Enteric‐type thymic adenocarcinoma shares similar immunohistochemical features with gastrointestinal epithelia [5, 19] and similar histopathology with primary gastrointestinal adenocarcinoma [20]. Oxaliplatin/5‐fluorouracil/leucovorin (FOLFOX) chemotherapy was first utilized by Gao et al., who successfully controlled the disease of a patient with metastatic enteric‐type thymic adenocarcinoma refractory to first‐line carboplatin/docetaxel, and second‐line nab‐paclitaxel/cisplatin+pembrolizumab [21] FOLFOX is widely used in the systemic treatment of unresectable, advanced or metastatic colon adenocarcinoma. FOLFIRINOX, which adds the topoisomerase inhibitor irinotecan to the FOLFOX regimen, is also used in these clinical settings. FOLFOX is also utilized in the neoadjuvant setting for proficient mismatch repair (pMMR) colon cancer, and in the adjuvant setting for both pMMR and deficient mismatch repair (dMMR) disease [22] In pancreatic adenocarcinoma, FOLFIRINOX is used in the neoadjuvant and first‐line settings for locally advanced disease, while FOLFOX may be used as first‐line systemic therapy for metastatic disease in patients with an intermediate performance status [23] Additionally, FOLFOX serves as a second‐line option for unresectable or metastatic biliary tract adenocarcinoma [24] For gastric adenocarcinomas, FLOT, which incorporates docetaxel alongside the FOLFOX regimen, is administered as a perioperative chemotherapy [25] With established utility in gastrointestinal adenocarcinomas, FOLFOX may present as a potential therapeutic option for this rare thymic malignancy.

In this case report, we describe a patient with unresectable enteric‐type thymic adenocarcinoma who demonstrated a good treatment effect on FOLFOX chemotherapy as first‐line therapy at the Oncology department at Eastern Health, Melbourne. The patient demonstrated a sustained treatment response through four cycles of treatment, maintaining her response during a 2.5‐month interruption between cycles 3 and 4, and maintaining stable disease for 6 months after electing to discontinue treatment after cycle 4 due to functional decline. The patient died from her disease 2 months after disease progression. This case represents the 50th reported case of this malignancy to date and the first reported case at an Australian institution. To our knowledge, this is the first reported case where FOLFOX was used as the first‐line systemic treatment of enteric‐type thymic adenocarcinoma.

## 2 Case Report

A 65‐year‐old woman presented to our department (Box Hill Hospital, Eastern Health, Melbourne) in May 2024 with progressive symptoms of thoracic outlet syndrome over 3 weeks, including neck discomfort, bilateral upper limb mottling on arm raise, and orthopnoea. She was otherwise well, with no infectious symptoms. She had a history of gastro‐oesophageal reflux disease, a hiatus hernia, a multinodular goitre, and a 15‐pack‐year smoking history. On initial examination, she had a plethoric appearance. She was afebrile, with a blood pressure of 120/62 mmHg (Reference range (RR): < 130 mmHg systolic, < 85 mmHg diastolic) [26], respiratory rate of 22 breaths/min (RR:12–20) [26], and peripheral oxygen saturation of 96% on room air (RR: 95%–100%) [26] with intermittent desaturation to 85% when lying flat. She demonstrated a positive Pemberton's sign and diminished heart sounds on auscultation. Her chest was clear to auscultation, and she was clinically euvolemic.

**FIGURE 1:**
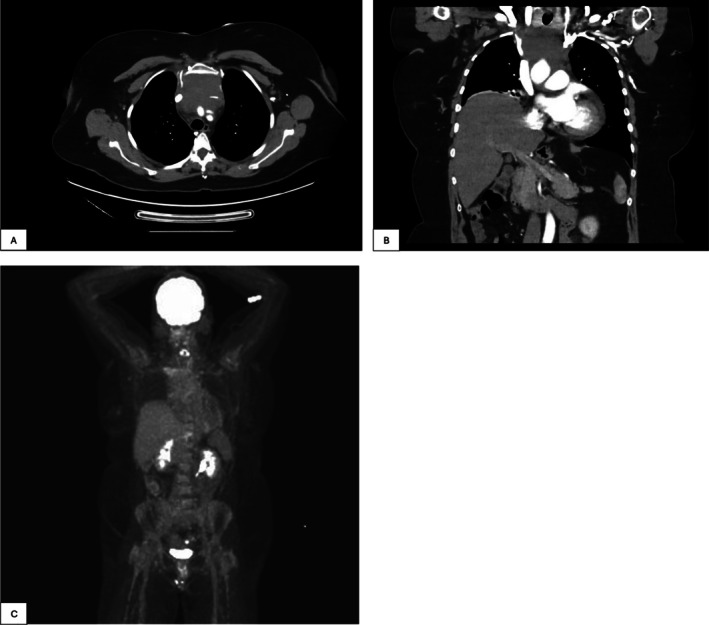
Initial CT and FDG PET imaging. (A) Initial CT: axial view. The tumor measures up to 6.0 cm in the anterior–posterior dimension. (B) Initial CT: coronal view. The tumour measures up to 7.5 cm in the transverse dimension and 6.0 cm in the superior–inferior dimension. (C) Initial PET: Maximum intensity projection.

A contrast‐enhanced staging computed tomography (CT) scan of the brain, neck, chest, abdomen, and pelvis showed a mild to moderate pericardial effusion with a thickness of up to 1.5 cm, and an anterior mediastinal soft tissue density measuring up to 7.5 × 6.0 × 6.0 cm (transverse × Anterior–Posterior × Superior–inferior) causing a lateral displacement and narrowing of the superior vena cava (SVC) and completely encasing, effacing, and obstructing the left brachiocephalic vein without evidence of distant sites of involvement (Figure 1A,B). Several low‐density rounded liver lesions were noted, measuring up to 1.8 cm in diameter, consistent with simple cysts on a subsequent liver ultrasound. A [18F] fluorodeoxyglucose positron emission tomography (FDG PET)‐CT was performed, whereby the mass in the anterior mediastinum demonstrated a moderately prominent diffusely homogeneous activity, similar to the background activity within the liver (Figure 1C). No potential organ or bone metastases were identified on PET‐CT. After a multidisciplinary team review, the anterior mediastinal mass was deemed surgically unresectable due to its encasement of the left brachiocephalic vein.

**FIGURE 2:**
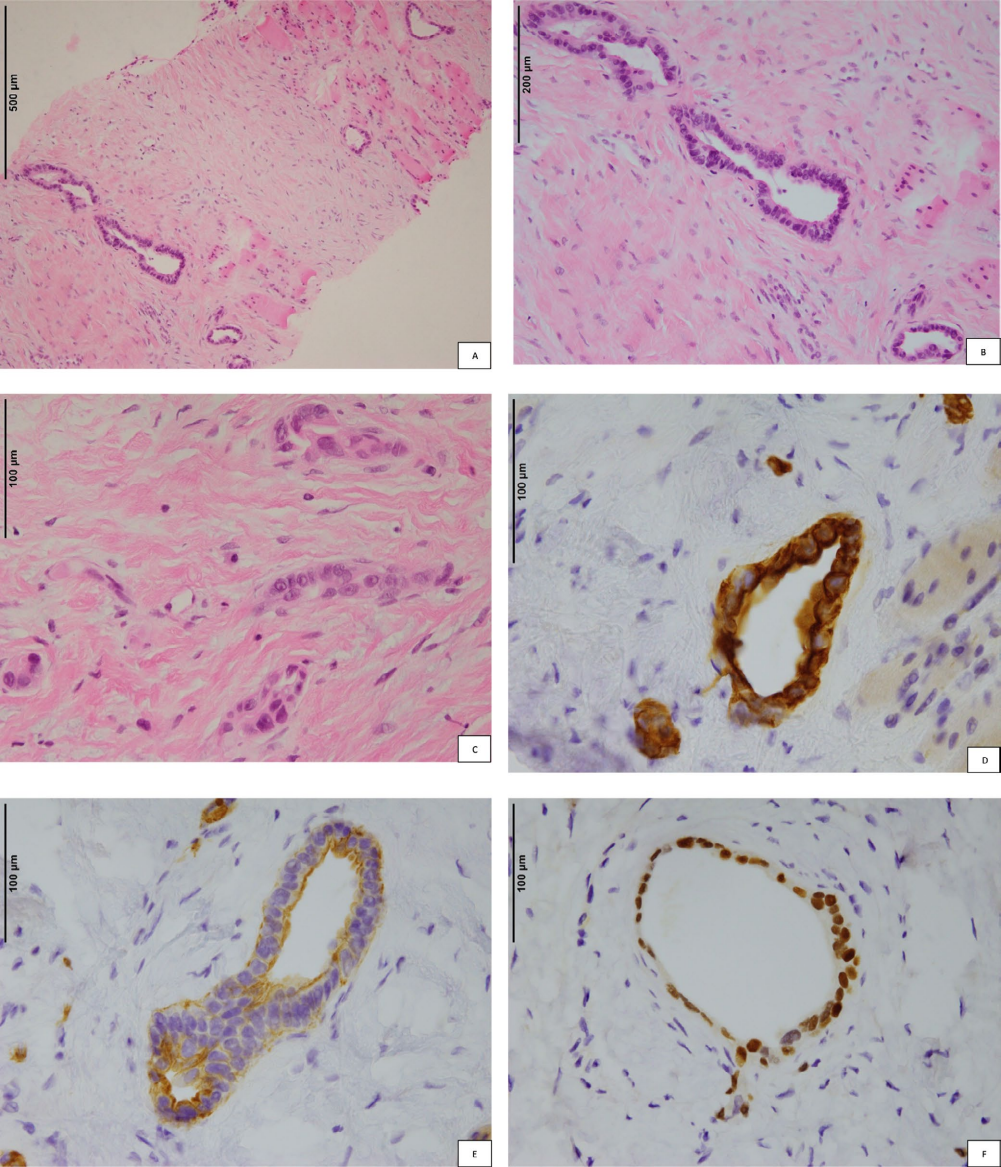
Histology and immunohistochemistry. (A) H&E‐stained section, original magnification ×100. Infiltrative glands lined by atypical columnar epithelium invading through fibrous stroma and adjacent muscle consistent with adenocarcinoma. (B) H&E‐stained section, original magnification ×200. Sections show glands lined by atypical columnar epithelial cells within fibrous stroma. (C) H&E‐stained section, original magnification ×400. Atypical glands lined by moderately pleomorphic columnar epithelium with vesicular nuclei, prominent nucleoli, and scattered mitoses (not pictured). (D) CK20 stain, original magnification ×400. Diffuse strong cytoplasmic staining for CK20 in all tumor cells, while CK7 showed only very patchy positive staining, favoring an enteric/lower gastrointestinal tract immunophenotype. (E) CD5 stain, original magnification ×400. Positive membranous staining for CD5 in atypical glandular epithelial cells favors a thymic origin. While no single immunohistochemical stain is specific for thymic carcinoma, the majority of cases will stain with at least one marker from a panel of CD5, C‐kit, and Pax‐8. (F) CDX‐2 stain, original magnification ×400. Positive nuclear staining in all tumor cells for CDX‐2. Strong diffuse staining with this marker is consistent with an enteric immunophenotype.

A CT‐guided core biopsy of the anterior mediastinal mass was performed in May 2024. The core biopsies showed fibrous tissue infiltrated by a moderately differentiated adenocarcinoma composed of tubular glands and clusters of columnar cells with pleomorphic hyperchromatic nuclei, prominent nucleoli, and occasional mitoses (Figure 2A–C). No thymic cysts were identified in the core biopsy. All immunostains were performed on a Leica Bond Prime automated stainer using standard protocols and positive controls. Immunostaining noted the following: CK7: occasional cells positive (Figure 2D), CK20: diffusely positive (Figure 2D), CD5: positive (Figure 2E), CDX2: patchy positive staining (Figure 2F), and CEA: patchy positive staining. Other immunostains, including markers for pulmonary (TTF‐1), gynaecological (PAX‐8/WT‐1/oestrogen receptor/progesterone receptor), and breast (GATA‐3), were negative or non‐contributory. C‐Kit, SATB2, and Calretinin were negative. There was, at minimum, focal staining of all four MMR proteins (MLH1, PMS2, MSH2, MSH6) and mismatch repair deficiency was not observed. The initial serum CEA level was normal, at 1.2 ng/mL.

The patient's presentation was consistent with a grade two SVC obstruction, and she underwent stenting of her occluded brachiocephalic and subclavian veins. This, however, was complicated by stent thromboses, requiring thrombolysis, stent replacement, and ongoing anticoagulation. The stents subsequently re‐occluded, leading to pharyngeal wall oedema secondary to venous congestion, identified on nasal endoscopy. She was commenced on dexamethasone to good effect, which was slowly weaned over 2 months.

Histopathology was consistent with a primary thymic adenocarcinoma of the enteric type. She was considered too great an anaesthetic risk to safely undergo an endoscopic workup to rule out a primary gastrointestinal malignancy. Following an internal Radiation Oncology meeting, a decision was made for the patient to undergo five fractions of 20 Gray palliative radiotherapy to her mediastinal mass in June 2024 to help relieve symptoms of her SVC syndrome. While chemoradiation was considered, it was decided that she would start on two‐weekly FOLFOX chemotherapy in the same month, without concurrent definitive radiation therapy. Definitive radiation was not initiated as she was still clinically unwell and recovering from her SVC syndrome, whereby the potential toxicities from definitive radiotherapy at a higher dose of 60–70 Gray over a minimum of 6 weeks would compound upon the toxicities of an already cytotoxic chemotherapy regimen. Furthermore, the patient resided at home alone and would have found it difficult to make the daily commute to and from the radiotherapy centre with no mode of transport. The radiation oncologists had planned to reassess her 1 month after her initial palliative radiotherapy to consider another session of palliative radiation. They also remained open to considering definitive radiotherapy at any stage, should the patient's functional status and prognostic trajectory improve on chemotherapy. FOLFOX, a systemic regimen commonly used in gastrointestinal adenocarcinomas, was selected due to the enteric characteristics of the patient's tumour. It was selected in preference over the then‐standard NCCN‐recommended regimen of carboplatin/paclitaxel for thymic carcinoma, which lacked specific trial evidence supporting its efficacy in thymic adenocarcinoma, particularly those of the enteric type. Given the heterogeneity of thymic carcinoma [27] a more specific approach to the enteric characteristics with FOLFOX was considered appropriate. Prior to her scheduled follow‐up with the Radiation Oncology team in July 2024, she became acutely unwell with a bacteraemia, resulting in a 2.5‐month interruption of her chemotherapy. She was not considered for additional radiotherapy during this period. Soon after resuming her fourth cycle of chemotherapy in early October 2024, she decided to discontinue any further active treatment due to her declining functional status.

**FIGURE 3:**
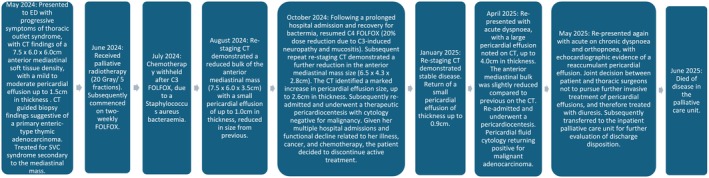
Timeline of patient events.

She had undergone three cycles before her chemotherapy was temporarily withheld in July 2024 due to a *Staphylococcus aureus* bacteraemia from her PICC line site, requiring a prolonged hospital admission for intravenous antibiotic therapy. During this hospital admission, she was re‐staged in August 2024, where she demonstrated a reduced bulk of her anterior mediastinal mass with dimensions of 7.5 × 6.0 × 3.5 cm (transverse × Anterior–Posterior × Superior–inferior). Her pericardial effusion measured up to 1.0 cm in thickness, reduced in size from her previous scan in May 2024. She recommenced cycle 4 FOLFOX in October 2024 after 2.5 months off chemotherapy, at a 20% dose reduction due to Cycle 3 FOLFOX‐induced neuropathy and mucositis. She was restaged again 10 days after Cycle 4 in October 2024, with CT demonstrating a further reduction in the size of the anterior mediastinal mass, measuring 6.5 × 4.3 × 2.8 cm (transverse × Anterior–Posterior × Superior–inferior) following cycle 4 FOLFOX. The CT, however, identified a marked increase in pericardial effusion size of up to 2.6 cm in thickness, to which the patient was readmitted to hospital and her pericardial effusion was treated to good effect with a pericardiocentesis. The pericardial fluid cytology later returned negative for malignancy. Her chemotherapy was deferred due to this hospital admission. Due to her multiple hospital admissions, intercurrent illness, and chemotherapy, the patient experienced a gradual functional decline. Following her hospital admission, she chose to discontinue chemotherapy indefinitely, citing fatigue and functional deterioration. A restaging CT in January 2025, performed 3 months after her last chemotherapy (cycle 4) and her previous restaging scan, showed stable disease, with a small pericardial effusion of thickness up to 0.9 cm that had returned. In April 2025, she was re‐admitted to the hospital with acute dyspnoea, where a CT identified a large pericardial effusion of up to 4.0 cm in thickness. The size of her anterior mediastinal mass on the CT was slightly reduced from her previous scan in January 2025. She underwent another pericardiocentesis, whereby her cytology results then returned positive for malignant adenocarcinoma. She re‐presented to the hospital in May 2025 with acute on chronic dyspnoea and orthopnoea in the setting of another reaccumulated pericardial effusion. She was treated partially with diuresis, and in discussion with the thoracic surgeons, the patient opted not for repeated invasive treatment of her pericardial effusion. In the setting of her chronic dyspnoea and functional acopia at home, she was discharged to the inpatient palliative care unit for further evaluation of disposition in May 2025. She deteriorated acutely whilst an inpatient at the palliative care unit and died in June 2025. A timeline of our patient's clinical progress is summarised in Figure 3.

## 3 Discussion

Differential diagnoses to consider in the evaluation of an anterior mediastinal mass include germ cell tumors, lymphomas, benign and malignant growths of the thymus and thyroid, and, less commonly, parathyroid adenomas, hemangiomas, sarcomas, and fibrosing mediastinitis. Furthermore, it is important to consider whether the mass originates from the mediastinum, the adjacent lung, or a different primary organ [28]. Thymic carcinomas are rare cancers with an incidence between 0.07 and 0.38 per 100,000 person‐years [29]. Thymic carcinomas are often large and invasive at the time of diagnosis, with tumor size at diagnosis averaging between 5.4 and 8.7 cm (range 2.1–19 cm), with common presentations including chest pain, dyspnea, and SVC syndrome from mediastinal mass effect [29]. SVC syndrome is a clinical diagnosis characterized by the symptoms and signs of SVC obstruction. In recent years, the immediate management of life‐threatening SVC obstruction has transitioned from that of radiotherapy to endovascular therapy [30]. While steroids are commonly used to treat airway compromise in SVC syndrome, [30] steroids can produce a cytotoxic effect on lymphoma cells, which may impair the diagnostic accuracy of a potential lymphoma in a patient presenting with a mediastinal mass, [31] should they be given steroids before a biopsy is conducted. As our patient's presentation of dyspnea in the setting of her SVC syndrome was subacute, and noting that she was vitally stable on first presentation, immediate administration of steroids was avoided until a diagnostic biopsy had been performed. Upon notice of posterior pharyngeal wall edema representing a high risk for airway compromise, however, steroid therapy was initiated promptly.

Enteric‐type thymic adenocarcinoma, as described in our patient, is a subtype of thymic adenocarcinoma exhibiting the expression of at least one of the following intestinal markers: CK20, CDX2, or MUC2 [4]. While the mechanism by which enteric‐type primary thymic adenocarcinomas express intestinal markers is unclear, it is plausible that these markers may have been present in some thymic cells from embryonic development, noting that the thymus and the gut‐derived organs arise from the same endodermal gut tube [32]. *CDX2* is a caudal‐type homeobox gene that encodes a transcription factor crucial for the proliferation and differentiation of intestinal epithelial cells. The CDX2 protein is typically expressed in the nuclei of these cells, spanning from the proximal duodenum to the distal rectum [33, 34]. MUC2 is an important gel‐forming mucin in the formation of gastrointestinal mucus [35]. CK20 is a cytokeratin intermediate filament with a high expression in normal glands and also in epithelial tumors of the gastrointestinal tract, urothelium, and Merkel cells [33]. CDX2 is expressed in almost all colorectal and bladder adenocarcinomas, while CK20 is expressed in almost all colorectal carcinomas and is often expressed in pancreatic, bile duct, urothelial, and gastric carcinomas [5]. Other markers we stained for included CK7, CD5, and TTF‐1. CK7 is another cytokeratin expressed in various glandular epithelia and epithelial tumors. CK7 is expressed in most adenocarcinomas; however, it is not classically expressed in primary tumors of the colon, thymus, prostate, kidneys, carcinoid tumors, and Merkel cell skin tumors [36, 37]. The CK7‐/CK20+ immunoprofile often suggests an adenocarcinoma of a primary gastrointestinal origin [33] and may assist in the differentiation of a primary enteric‐type thymic adenocarcinoma from a gastrointestinal metastatic deposit.

CD5 is a surface glycoprotein receptor classically expressed on thymocytes, mature T lymphocytes, and in some B‐cell lymphomas, including chronic lymphocytic leukemia and mantle cell lymphoma [38, 39]. CD5 expression serves as a negative regulator of T cell activation in thymocyte development [40]. CD5 is often expressed in thymic carcinoma, [1] and is very rarely expressed in malignancies with a gastrointestinal primary, thereby serving as a useful marker in discerning primary thymic carcinoma from metastatic disease seeding into the thymus [38, 41]. CD5 expression also has some utility in differentiating thymic carcinomas from other anterior mediastinal malignancies. In the analysis of 100 cases of anterior mediastinal neoplasms, Saad et al. ascribed a sensitivity and specificity of 90% and 100% respectively, to CD5 in distinguishing thymic carcinoma from thymoma, and a sensitivity and specificity of 90% and 60%, respectively, in the application of CD5 to distinguish between thymic carcinoma and other anterior mediastinal tumors [42]. It should be noted, however, that while expressional studies have identified a pattern of CD5 expression in thymic carcinomas, most data derive from thymic squamous cell carcinomas or unspecified thymic carcinoma types [38, 42, 43]. CD5 is expressed in over 90% of thymic squamous cell carcinomas, and although it is not characteristically associated with most thymic adenocarcinoma subtypes, it has demonstrated positive expression in 10%–50% of enteric‐type thymic adenocarcinomas [44]. Although our patient's histopathology identified an adenocarcinoma rather than a squamous cell carcinoma, the positive staining for CD5 in her tissue supports a primary thymic malignancy rather than one of gastrointestinal origin. This is based on the understanding that CD5 is physiologically expressed in developing thymocytes, its unlikely expression in primary gastrointestinal malignancies, and its positive expression in 10%–50% of enteric‐type thymic adenocarcinomas.

TTF‐1 is a transcription factor classically expressed in cells derived from the foregut endoderm and neuroectoderm, including type II alveolar epithelial cells and thyroid follicular cells. TTF‐1 is an important diagnostic marker in primary thyroid and lung malignancies [45], with TTF‐1 holding a sensitivity of up to 84% and specificity of 85%–100% in identifying lung adenocarcinoma [46] Therefore, TTF‐1 has diagnostic utility in the workup of a new mediastinal mass, in differentiating primary lung and thyroid malignancies from other malignancies.

Our patient was given a diagnosis of primary thymic adenocarcinoma of the enteric type, due to the positivity of her tissue biopsy for the enteric markers CK20 and CDX2. The CD5 positivity was suggestive of a primary thymic malignancy, and radiologically, there was no evidence of a gastrointestinal primary. Furthermore, her serum CEA level has been consistently within normal limits since initial presentation, whereby CEA is a nonspecific tumour marker holding a close association with colorectal cancer, while also often being expressed in gastric, pancreatic, lung, renal, and breast cancers [47, 48, 49]. While a gastroscopy and colonoscopy would have been ideal to more definitively exclude a gastrointestinal primary, our patient was medically unstable and considered too great an anaesthetic risk for endoscopic investigation at initial presentation. She remained medically unstable in the following months, during her prolonged hospitalisation for a bacteraemia, and again during her October 2024 readmission with a large reaccumulation of her pericardial effusion. Later in October 2024, when a decision was made by the patient to cease active treatment, endoscopy was deemed no longer necessary, as it had no implications on her management, and the focus had shifted to best supportive care.

The Masaoka staging system is commonly used for the staging of thymic malignancies, including thymic carcinomas and thymomas. A distinction should be drawn between thymic carcinomas and thymomas. Thymomas are neoplasms originating from thymic epithelial cells, often demonstrating a differentiation toward thymic epithelial cells and containing a variable proportion of non‐neoplastic lymphocytes. Thymic carcinomas, while also malignant epithelial tumors, are characterized by greater invasiveness and lack the thymus‐like features of thymomas [50].

Radiological imaging at presentation in May 2024 showed no evidence of pleural, lymphatic, or hematogenous metastases. However, a moderate pericardial effusion was present, initially attributed to her SVC syndrome. Her disease on presentation was thought to be early‐stage or locally advanced. Due to the surgical unresectability of our patient's mediastinal mass, we were unable to obtain a whole tumor specimen to assess for microscopic or macroscopic thymic transcapsular invasion or macroscopic invasion into a neighboring organ, and were unable to ascribe an exact Masaoka stage at diagnosis. The pericardial effusion improved on her restaging scan in August 2024, but recurred multiple times since, requiring intervention. Her pericardial fluid cytology was negative for malignancy in October 2024; however, in April 2025, the fluid cytology was positive for adenocarcinoma, confirming pericardial metastasis and classifying her disease then as Masaoka‐Koga stage IVa, or T2NxM1/stage IVA.

While surgical resection remains the mainstay of treatment for early‐stage and locally advanced thymic carcinomas [29] the anatomical proximity of an anterior mediastinal mass to the heart, great vessels, and airways can pose great anaesthetic and surgical risks, including the potential for acute haemodynamic or respiratory compromise [51] Per the NCCN guidelines, locally advanced unresectable thymic carcinomas are recommended for treatment with concurrent chemoradiation [7] Under these guidelines, the preferred first‐line chemotherapy regimen is carboplatin/paclitaxel+ramucirumab with concurrent radiation therapy for thymic carcinoma, and this regimen encompasses all subtypes of thymic carcinoma in these guidelines. Given the close immunohistochemical resemblance of enteric‐type thymic adenocarcinoma to colorectal malignancies, Gao et al. have noted that FOLFOX regimens may also be appropriate for first‐line chemotherapy in unresectable disease [21] In colon cancer with pMMR, fluoropyrimidine‐based chemotherapy is a staple of treatment in unresectable or metastatic disease, and in both neoadjuvant and adjuvant settings in resectable disease [22] Fluoropyrimidine‐based chemotherapy is not commonly administered concurrently with radiotherapy in colon cancer. However, in certain cases, such as initially unresectable T4 colon cancer, neoadjuvant radiotherapy may be considered alongside fluoropyrimidine‐based chemotherapy to convert the tumour into a resectable state [22] Leucovorin, a folate analogue [52] is coupled with 5‐Fluorouracil (5‐FU), a fluoropyrimidine chemotherapeutic agent, to serve as a chemoprotectant, creating a stable complex to potentiate the effects of 5‐FU [52] and mitigating its side effects [53] 5‐FU+leucovorin+oxaliplatin (FOLFOX) is a common first‐line treatment for unresectable pMMR colon cancer [22] In our patient, who had pMMR disease, palliative radiation was initiated for SVC syndrome, followed by first‐line FOLFOX chemotherapy. Our patient responded well to FOLFOX, despite a 2.5‐month interruption between Cycles 3 and 4. She maintained stable disease for 6 months after treatment cessation, before developing disease progression with evidence of pericardial metastases in April 2025, and dying of disease in June 2025.

There are 35 case reports in the literature describing 49 cases of primary enteric‐type thymic adenocarcinoma. There are 44 cases where patient treatment was documented (Table 1). Of note, in Table 1, while patients for whom their case reports were described as receiving a ‘tumour resection’ likely received this via a thymectomy, this is unconfirmed as the corresponding case reports do not specify this. ‘WNL’ describes the corresponding tumour marker to be within normal limits; however, the exact concentration is not given by the corresponding case report. Not all case reports have documented the fractions and number of Gray used in radiotherapy treatments, nor the number of cycles of chemotherapy given. Certain positive markers are not given a degree of positivity by the corresponding case report. These have been ascribed as ‘+’ only in Table 1. Within Table 1, 22 case reports [6, 20, 55, 56, 59, 61, 62, 63, 65, 66, 67, 68, 69, 70, 71, 72, 73, 74, 75, 76, 77, 78] describe 32 cases of thymic adenocarcinoma that were not explicitly labelled as ‘enteric‐type’; however, these cases can be classified as enteric‐type thymic adenocarcinoma based on their positivity for the enteric markers CDX2, CK20, or MUC2. Quevedo et al. [79], Abu salah et al. [80], Conforti et al. (case 4) [60], Shiono et al. [81], and Weissferdt et al. [82] each described a case of enteric‐type thymic adenocarcinoma that lacked treatment information, or had not been initiated on cancer treatment, and thus were not included in Table 1.

**TABLE 1:** Updated record of reported cases of primary enteric‐type thymic adenocarcinoma with documented treatment, in reverse chronological order.

Case	Age, Sex	Presentation	Diagnosis, Masaoka stage	CDX2	CK20	MUC2	CD5	CK7	TTF‐1	Gastrointestinal workup	Treatment/response	Overall survival
Matias‐Cruz et al. [54]	54F	Headaches, dizziness, chest pain	Enteric‐type thymic adenocarcinoma, stage: NA	+	+	NA	+	−	−	PET‐MRI: No potential gastrointestinal primary found Gastrointestinal Tumour markers: CEA: 5600 ng/mL (reference < 5 ng/mL), KIT	‐ Deemed unresectable following diagnostic CT in May 2021. ‐ Underwent a percutaneous biopsy of the mediastinal mass in June 2021 and commenced on induction CTx soon after return of results. ‐ Induction carboplatin/paclitaxel CTx for 4 cycles‐ Partial response on subsequent PET‐CT, however still unresectable. ‐ Subsequent carboplatin/paclitaxel CTx+mediastinal RTx 30 Frx 60Gy. Treatment completed in January 2022, with partial reduction in tumour size at 20 months.	20 months (APR)
Gao et al. [21]	38M	Chest discomfort	Enteric‐type thymic adenocarcinoma, stage: IVb	+	+	NA	NA	+	−	Gastrointestinal endoscopy, CT: No potential gastrointestinal primary found Gastrointestinal tumour markers: NA	‐ Following return of biopsy results, received carboplatin/docetaxel CTx for 6 cycles, with no significant change in mediastinal mass on subsequent repeat CT. ‐ Subsequent RTx with slight reduction of mass size. ‐ On restaging PET‐CT 9 months after initial diagnosis, noted new metastasis of cerebellum and disease progression in known psoas muscle metastasis. ‐ Patient then received RTx for the brain, psoas major, lumbar vertebral metastases+Nab‐paclitaxel/cisplatin CTx+pembrolizumab for 3 cycles with minimal effect. ‐ Then received FOLFOX CTx+cetuximab for 4 cycles ‐ Repeat Lumbosacral MRI showed a significant reduction in number of muscle metastases.	14 months (APR)
O'Shea et al. [55]	55M	Chest discomfort, fever, fatigue	Thymic adenocarcinoma, stage: I	+	+	NA	NA	NA	−	PET‐CT: No potential gastrointestinal primary found Gastrointestinal tumour markers: CA19‐9, CEA, AFP WNL	‐ Noting initial leukocytosis and raised peripheral blast count, received a bone marrow aspirate diagnosing high‐risk acute myeloid leukaemia ‐ Subsequent staging CT identifying an anterior mediastinal mass, which was biopsied showing positivity for enteric markers; however, no organ primary was identified. Repeat biopsy also did not identify organ primary of mediastinal malignancy. ‐ Subsequently received induction Idarubicin/cytarabine CTx+midostaurin to treat high‐risk FLT 3 acute myeloid leukaemia. Subsequent repeat bone marrow biopsy showed complete remission. ‐ After a 4‐week CTx holiday, underwent a thymectomy with the classification of Masaoka Stage 1 thymic adenocarcinoma. ‐ Commenced on consolidation CTx 4 weeks post thymectomy due to concurrent high‐risk AML. Has also received an allogenic haematopoietic stem cell transplant. ‐ Well at report publication, > 3 months post‐operatively.	> 3 months (A)
Hamanaka et al. [3]	53F	Left hilar tumour shadow on CXR	Enteric‐type thymic adenocarcinoma, stage: II	+	+	NA	−	−	−	Gastroscopy+colonoscopy+internal capsule endoscopy, PET‐CT: No potential gastrointestinal primary foundGastrointestinal tumour markers: CEA: 2.7 ng/mL, KIT—	‐ Thymectomy+adjuvant RTx 25 Frx 50Gy. ‐ Recurrence‐free at 6 months post‐operatively.	6 months (ACR)
Ersoz et al. [56]	55M	Dysphagia, right shoulder pain	Moderately differentiated non‐mucinous thymic adenocarcinoma, stage: III	Focal+	−	NA	Focal+	Diffuse+	−	Gastroscopy+colonoscopy, PET: No potential gastrointestinal primary found Gastrointestinal tumour markers: CEA diffuse+, KIT−	‐ Tumour resection+right lung wedge resection (due to adherence of mediastinal mass to right lung). ‐ Adjuvant RTx with 54 Gy IMRT. ‐ Two new cranial metastases identified on MRI 6 months post‐operatively. ‐ Subsequently received 22Gy SRS, followed by carboplatin/Paclitaxel CTx. ‐ After 4 cycles of CTx, at 12 months post tumour resection, the patient had MRI findings of complete resolution of the two metastases, however two new brain metastases were found. ‐ Subsequently received 18Gy SRS, and CTx regimen was changed to doxorubicin/cyclophosphamide/cisplatin CTx. ‐ At 17 months post‐operatively, regression of cranial disease noted on repeat MRI Brain.	28 months (ACR)
Li et al. [57]	44F	Dyspnoea, chest pain	Enteric‐type thymic adenocarcinoma, stage: IVb	+	+	NA	NA	−	−	PET‐CT: No potential gastrointestinal primary found Gastrointestinal tumour markers: CEA: 20.07 ng/mL, CA19‐9: 483.98 U/mL (reference < 37 U/mL), CA125: 111.44 IU/mL (reference < 35 IU/mL), CA242: 138.50 U/mL (reference < 20 IU/mL), CK19: 5.34 ng/mL (reference < 3.3 ng/mL)	‐ XELOX for 6 cycles. ‐ RTx 30Fr × 60Gy given concurrently during first 3 CTx cycles. ‐ Omitted the use of apatinib (VEGFR2 inhibitor) due to side effects of myelosuppression and radioactive oesophagitis. ‐ Reduction of thymic mass size noted on subsequent re‐staging CT after C6 XELOX.	16 months (APR)
Ishida et al. [58]	39F	Nail clubbing, bilateral hand edema	Enteric‐type thymic adenocarcinoma, stage: NA	Lesion 1: − Lesion 2: partial+	Lesion 1: − Lesion 2: −	Lesion 1: − Lesion 2: −	Lesion 1: + Lesion 2: +	NA	Lesion 1: − Lesion 2: −	FDG‐PET, CT: No potential gastrointestinal primary found Gastrointestinal tumour markers: Lesion 1: KIT−, SATB2¬ Lesion 2: KIT−, SATB2 partial+	‐ Tumour resection+partial resection of left upper lung lobe (due to invasion of lesion 1 (solid mass) into lung lobe). ‐ Adjuvant RTx 25 Fr × 50Gy. ‐ Disease‐free at approximately 2 years.	2 years (ACR)
Himuro et al. [59]	58F	Treated for a left cerebellar tumour. Subsequent chest CT identifying an anterior mediastinal mass.	Mucinous thymic adenocarcinoma, stage: IVb	+	Focal+	NA	−	Focal+	−	Gastroscopy+colonoscopy, CT, FDG‐PET: No potential gastrointestinal primary foundGastrointestinal tumour markers: CEA+, KIT—	‐ Surgical resection of the left cerebellar metastasis. ‐ Subsequently underwent thymectomy+resection of the pericardium and left phrenic nerve, and lymphadenectomy due to nodal metastasis. Microscopic residual disease was noted. ‐ Originally planned for adjuvant mediastinal RTx however a new cerebellar metastasis was found adjacent to the site of the cerebellar lesion resection 1‐month post‐ thymectomy, to which the patient underwent gamma knife radiosurgery and RTx over the site of the new cerebellar lesion first. ‐ However, the cerebellar tumour continued to progress, whereby the patient DOD 6 months post‐thymectomy.	6 months (DOD)
Hamahiro et al. [20]	68M	Pericardial effusion	Mucinous thymic adenocarcinoma, stage: IVb	+	+	NA	−	−	−	Review of organ systems: No potential gastrointestinal primary found Gastrointestinal tumour markers: CEA: 69.2 ng/mL	‐ Pericardiocentesis performed, with pericardial fluid cytology returning as adenocarcinoma. ‐ Subsequent CT identifying an anterior mediastinal mass with pericardial invasion, mediastinal lymphadenopathy, bilateral pleural effusion, pleural metastases and a liver metastasis. ‐ Underwent a biopsy of the mediastinal tumour and pleural metastasis. ‐ Mediastinal tumour deemed unresectable. ‐ Received carboplatin/paclitaxel CTx with subsequent PD and switched to carboplatin/pemetrexed CTx however DOD 6 months post‐operatively due to continued PD.	6 months (DOD)
Conforti et al. [60] Case 1	44M	NA	Enteric‐type thymic adenocarcinoma, stage: III	+	+	NA	Focal+	−	NA	NA	‐ Received carboplatin CTx+RTx with PD at 9 months ‐ Subsequent surgery+RTx+sunitinib ‐ AWD at 19 months.	19 months (AWD)
Conforti et al. [60] Case 2	60M	NA	Enteric‐type thymic adenocarcinoma, stage: IVb	Focal+	Focal+	NA	NA	−	NA	NA	‐ Received carboplatin/paclitaxel CTx with PD at 5 months. ‐ Subsequent capecitabine/irinotecan CTx. ‐ DOD at 16 months.	16 months (DOD)
Conforti et al. [60] Case 3	24M	NA	Enteric‐type thymic adenocarcinoma, stage: IVb	+	+	NA	NA	Focal+	NA	Gastrointestinal tumour markers: CEA WNL	‐ Received cisplatin+ etoposide CTx+RTx with PD at 9 months. ‐ Subsequent RTx+cisplatin/doxorubicin/cyclophosphamide CTx. ‐ DOD at 4 years.	4 years (DOD)
Tamai et al. [41]	29F	Abnormal chest shadow on CXR as a part of a routine medical	Enteric‐type thymic adenocarcinoma, stage: NA	Mucinous cells: + Non‐mucinous cells: +	Mucinous: + Non‐mucinous: −	Mucinous: − Non‐mucinous: −	Mucinous: − Non‐mucinous: −	Mucinous: focal+ Non‐mucinous: +	Mucinous: ‐ Non‐mucinous: −	FDG‐PET: No potential gastrointestinal primary found Gastrointestinal tumour markers: Mucinous cells: KIT−. MUC6− Non‐mucinous cells: KIT‐, MUC6: focal+	‐ Thymectomy. ‐ Recurrence‐free at 8 months.	8 months (ACR)
Narvaez Muñoz et al. [61]	58M	Admitted with endocarditis. Incidental finding of mediastinal mass on preoperative CT.	Mucinous thymic adenocarcinoma, stage: NA	+	+	NA	+	NA	NA	Gastrointestinal tumour markers: CA19‐9+	‐ Tumour resection+4Fr adjuvant RTx. ‐ Recurrence free at 1 year.	1 year (ACR)
Sakanoue et al. [62]	39F	Referred for investigation of an abnormal chest CT	Mucinous thymic adenocarcinoma, stage: III	+	+	NA	Focal+	+	−	Gastroscopy, colonoscopy, abdominopelvic US, other radiology: No potential gastrointestinal primary found Gastrointestinal tumour markers: CA19‐9: 80.5 U/mL	‐Tumour resection+resection of the pericardium, right phrenic nerve, right internal artery, left brachiocephalic vein due to tumour invasion, and partial resection and venoplasty with a pericardium patch for the superior vena cava with a polytetrafluoroethylene graft. ‐ Multiple lung metastases detected 1 year post operatively, to which the patient underwent CTx. ‐ Alive at 34 months post‐operatively.	34 months (A)
Kinoshita et al. [63]	79F	NA	Mucinous thymic adenocarcinoma, stage: NA	+	+	NA	NA	−	NA	Gastrointestinal endoscopy, CT, PET‐CT: No potential gastrointestinal primary found	‐ Thymectomy+resection of Right middle lung lobe. ‐ No adjuvant therapy given. ‐ Recurrence free at 20 months post‐operatively.	20 months (ACR)
Haruki et al. [64]	61F	Upper limb and facial oedema	Enteric‐type thymic adenocarcinoma, stage: NA	+	+	NA	NA	NA	NA	Systemic workup: No potential gastrointestinal primary found Gastrointestinal tumour markers: CEA: 7.1 ng/mL	‐ Weekly carboplatin/paclitaxel CTx+RTx 25Frx 2.0Gy, with partial response after the induction chemoradiotherapy with a 43% reduction in the tumour diameter. ‐ Subsequently underwent tumour resection, with resection of part of the pericardium, part of the right lung upper lobe, both brachiocephalic veins, azygos vein, right phrenic nerve and superior vena cava due to tumour invasion, followed by SVC reconstruction with a polytetrafluoroethylene graft. No adjuvant CTx was undertaken. ‐ Disease‐free at 1 year post‐operatively.	ACR 1 year post‐operatively
Yin et al. [65]	44F	Anterior mediastinal mass detected on physical examination	Mucinous thymic adenocarcinoma, stage: NA	Diffuse+	Diffuse+	NA	NA	NA	−	Endoscopy, other radiology: No potential gastrointestinal primary found Gastrointestinal tumour markers: KIT+	‐ Surgical resection of tumour+adjuvant CTx 1 month post‐operatively. ‐ DOD 13 months post‐operatively.	13 months (DOD)
Lee et al. [66]	31M	Chest tightness and discomfort	Mucinous thymic adenocarcinoma, stage: NA	+	+	NA	Focal+	+	−	CT: No potential gastrointestinal primary found Gastrointestinal tumour markers: CEA: 37.5 ng/mL, CA19‐9: 64.9 U/mL, AFP WNL	‐ Doxorubicin/cyclophosphamide/cisplatin CTx, and then treated with etoposide/ifosfamide/cisplatin CTx. ‐ Thoracic and lumbar spine, mediastinal lymph node, and pelvic metastases developed 3 months after first treatment. The mediastinal mass also grew, with development of new lung metastases. ‐ Underwent laminectomy due to malignant cord compression caused by tumour extension at T10 and L2.	NA
Kwon et al. [67] case 1	56F	Cough, sputum production	Thymic adenocarcinoma NOS, stage: III	Focal+	+	Focal+	Focal+	−	−	CT, PET: No potential gastrointestinal primary found Gastrointestinal tumour markers: CEA WNL	‐ Docetaxel/cisplatin CTx. ‐ DOD at 5 years+3 months.	5 years+3 months (DOD)
Kwon et al. [67] case 2	46M	Nonspecific	Mucinous thymic adenocarcinoma and adenocarcinoma NOS, stage: III	+	+	Focal+	Focal+	−	−	CT, PET: No potential gastrointestinal primary found Gastrointestinal tumour markers: CEA elevated, CEA+	‐ Surgical resection+cyclophosphamide/doxorubicin/cisplatin CTx+RTx. ‐ DOD at 2 years+9 months.	2 years+9 months (DOD)
Kwon et al. [67] case 3	51M	Shoulder and back pain	Mucinous thymic adenocarcinoma, stage: IVb	+	NA	NA	−	NA	−	CT, PET: No potential gastrointestinal primary found Gastrointestinal tumour markers: CEA elevated	‐ RTx. ‐ AWD at 1 year+2 months.	1 year+2 months (AWD)
Kwon et al. [67] case 4	34M	NA	Mucinous thymic adenocarcinoma, stage: IVb	+	+	NA	−	Weak+	−	CT, PET: No potential gastrointestinal primary found Gastrointestinal tumour markers: NA	‐ Cyclophosphamide/doxorubicin/cisplatin CTx ‐ BAY86‐9766 (MEK inhibitor) trialled as second‐line therapy. ‐ AWD at 1 year.	1 year (AWD)
Kwon et al. [67] case 5	56F	Nonspecific	Mucinous thymic adenocarcinoma, stage: III	−	+	+	−	−	−	CT, PET: No potential gastrointestinal primary found	‐ Surgical resection ‐ PD at 1 year.	1 year+5 months (AWD)
Kwon et al. [67] case 6	70M	Chest wall pain	Mucinous thymic adenocarcinoma and adenocarcinoma NOS, stage: II	−	−	+	−	+	−	CT, PET: No potential gastrointestinal primary found Gastrointestinal tumour markers: NA	‐ Paclitaxel/cisplatin CTx+RTx. ‐ PD at 5 months.	8 months (AWD)
Kwon et al. [67] case 7	50M	Chest discomfort, weight loss	Thymic adenocarcinoma NOS, stage: IVb	+	+	Focal+	−	−	−	CT, PET: No potential gastrointestinal primary found Gastrointestinal tumour markers: NA	‐ Cyclophosphamide/doxorubicin/cisplatin CTx+RTx. ‐ PD at 5 months.	7 months (AWD)
Kwon et al. [67] case 8	62M	Chest discomfort	Mucinous thymic adenocarcinoma, stage: IVb	+	Focal+	Focal+	−	−	−	CT, PET: No potential gastrointestinal primary found Gastrointestinal tumour markers: CEA elevated	‐ Cyclophosphamide/doxorubicin/cisplatin CTx. ‐ AWD at 5 months.	5 months (AWD)
Kwon et al. [67] case 9	57F	Dyspnoea, rib pain	Mucinous thymic adenocarcinoma, stage: IVb	+	+	+	−	−	−	CT, PET: No potential gastrointestinal primary found Gastrointestinal tumour markers: CEA+	‐ Cyclophosphamide/doxorubicin/cisplatin CTx ‐ PD at 3 months.	5 months (AWD)
Sawaki et al. [68]	59M	Anterior chest swelling and pain	Thymic adenocarcinoma, stage: IVb	+	+	NA	+	−	−	Gastroscopy+colonoscopy: No potential gastrointestinal primary found Gastrointestinal tumour markers: CEA: 18.6 ng/mL, CEA+	‐ First line XELOX CTx. ‐ Palliative RTx to T10 metastasis for pain control at 10Fr × 30Gy during C1 and C2 of XELOX. ‐ Temporary decrease in right pleural effusion and improvement in serum biochemistry after Cycle 2 of XELOX, however these worsened prior to cycle 3. ‐ Zolendronic acid given to minimise pathological fracture risk. ‐ PD post C4 XELOX. ‐ As the tumour was KRAS wild type, the patient was switched to second line cetuximab+FOLFIRI CTx, with subsequent normalisation of LDH and improvement in ALP levels after one cycle. ‐ Interstitial pneumonia developed after C2 cetuximab+FOLFIRI CTx with suspicion of Pneumocystis jiroveci pneumonia, for which the patient was commenced on sulfamethoxazole+trimethoprim and steroids. ‐ Subsequently resumed single agent FOLFIRI without cetuximab with worsening functional status after one cycle and CTx was subsequently ceased. ‐ DOD at 6 months from diagnosis.	6 months (DOD)
Wang et al. [69]	70M	Chest tightness, dyspnoea, chest pain	Thymic adenocarcinoma, stage: NA	+	+	NA	−	+	−	Radiology: No potential gastrointestinal primary found Gastrointestinal tumour markers: CEA WNL, CA 19‐9 WNL, Villin+	‐ Tumour resection. ‐ No adjuvant therapy provided. ‐ Recurrence‐free at 7 months.	7 months (ACR)
Moser et al. [5] Case 1	41M	Asymptomatic	Enteric‐type thymic adenocarcinoma, stage: I	+	+	NA	−	NA	−	Gastroscopy+colonoscopy, CT: No potential gastrointestinal primary found Gastrointestinal tumour markers: CEA+, KIT−, CA19‐9 WNL, AFP WNL	‐ Thymectomy+resection of mediastinal fatty tissue. ‐ Due to complete resection of the tumour, no adjuvant therapy was provided. ‐ Recurrence‐ free at 18 months.	18 months (ACR)
Moser et al. [5] Case 2	39F	Asymptomatic	Enteric‐type thymic adenocarcinoma, stage: I	+	+	NA	Focal+	Focal+	−	Gastroscopy+colonoscopy, CT: No potential gastrointestinal primary found Gastrointestinal tumour markers: CEA+, KIT‐, CA19‐9 WNL, AFP WNL	‐ Tumour resection. ‐ Disease recurrence in anterior mediastinum at re‐staging 23 months after initial resection. No local or distant spread. The recurrent tumour was adherent to the pericardium. The recurrent tumour and affected adherent pericardium were surgically resected again at 24 months post initial tumour resection, without adjuvant treatment. ‐ Disease recurrence again at 39 months post resection of the local recurrence. No local or distant spread. Surgical resection was recommended again however the patient declined this.	159 months (AWD)
Jung et al. [70]	59F	Left lower quadrant pain	Thymic adenocarcinoma, stage: IVb	Diffuse+	Diffuse+	NA	+	Focal+	−	Gastrointestinal endoscopy, PET: No potential gastrointestinal primary found Gastrointestinal tumour markers: CEA: 8.73 ng/mL, CA19‐9: 252.2 U/mL	‐ Thymectomy with resection of pericardium (due to pericardial invasion). ‐ Unable to achieve complete resection due to invasion of the tumour into the pericardium, phrenic nerve, innominate vein and aorta. ‐ Adjuvant palliative CTx+RTx. ‐ Alive with aggravated bone and lung metastases at 11 months.	11 months (AWD)
Maghbool et al. [71]	28F	Neck and right upper extremity pain, dyspnoea	Thymic adenocarcinoma, stage: NA	Focal+	Focal+	NA	Diffuse+	Focal+	−	Endoscopy+colonoscopy, abdominopelvic sonography, other radiology: No potential gastrointestinal primary found Gastrointestinal tumour markers: CEA WNL, CA19‐9: 2420 U/mL, KIT−, AFP WNL, Villin+	‐ Tumour resection+Adjuvant GEMOX CTx+RTx. ‐ Disease‐free at 6 months.	6 months (ACR)
Teramoto et al. [72]	55M	Right lower lung shadow incidentally found on CXR from a routine health check	Papillo‐tubular thymic adenocarcinoma, stage: NA	+	+	NA	+	+	−	FDG‐PET, CT: No potential gastrointestinal primary found Gastrointestinal tumour markers: CEA WNL, CA19‐9 WNL, CK19 WNL	‐ Initial anterior mediastinal lesion on CT thought to be a thick‐walled thymic cyst and therefore not treated immediately. ‐ Repeat CT 5 months later showed growth of the mass suspicious for malignancy, to which the patient was admitted for treatment. ‐ Thymectomy+resection of adjacent pericardium (due to tumour attachment to pericardium)+adjuvant RTx 50Gy ‐ Recurrence free 14 months post‐operatively.	14 months (ACR)
Seon et al. [73]	66F	Angina	Mucinous thymic adenocarcinoma, stage: NA	NA	+	NA	Diffuse+	NA	NA	Radiology: No potential gastrointestinal primary found Gastrointestinal tumour markers: NA	‐ Myocardial SPECT identifying RCA territory ischemia. ‐ Coronary angiography identifying proximal RCA narrowing due to external compression by a mediastinal mass. ‐ Tumour resected. ‐ Recurrence‐free at 5 years.	5 years (ACR)
Abdul‐Ghafar et al. [74]	36F	Referred for further investigation and treatment of a mediastinal mass	Mucinous thymic adenocarcinoma, stage: NA	Focal+	Diffuse+	NA	Focal+	Diffuse+	−	Gastroscopy+colonoscopy, CT, PET: No potential gastrointestinal primary found Gastrointestinal tumour markers: NA	‐ Tumour resection+adjuvant RTx+paclitaxel/cisplatin CTx for 6 cycles. ‐ Disease recurrence with metastasis at 1 year followup.	15 months (DOD)
Maeda et al. [75] Case 1	52F	Parasternal bulge	Mucinous thymic adenocarcinoma, stage: NA	Diffuse+	Focal+	Diffuse+	Diffuse+	−	−	PET, CT: No potential gastrointestinal primary foundGastrointestinal tumour markers: CEA: 3.8 ng/mL, CEA+, MUC6—	‐ Thymectomy+resection of the sternum, left ribs, pericardium and left brachiocephalic vein. Microscopic residual disease noted. ‐ Adjuvant cisplatin/etoposide CTx+RTx. ‐ Developed right lung and cervical lymph node metastases at 7 months. ‐ Switched to carboplatin/paclitaxel CTx without effect. ‐ AWD at 11 months.	11 months (AWD)
Maeda et al. [75] Case 2	38M	Chest pain	Mucinous thymic adenocarcinoma, stage: IVa	Focal+	Diffuse+	−	Focal+	Focal+	−	Gastrointestinal endoscopy, CT, abdominal US, whole‐body gallium‐67 scintigraphy: No potential gastrointestinal primary found Gastrointestinal tumour markers: CEA+	‐ Tumour resection+resection of the superior vena cava, left brachiocephalic vein and pericardium. Macroscopic residual disease noted. ‐ Adjuvant carboplatin/docetaxel CTx+RTx. ‐ Subsequently developed bone metastases and a malignant pleural and pericardial effusion. ‐ DOD at 12 months.	12 months (DOD)
Maeda et al. [75] Case 3	55M	Chest tightness	Mucinous thymic adenocarcinoma, stage: IVb	Diffuse+	Diffuse+	Focal+	Focal+	−	−	Radiology: No potential gastrointestinal primary found Gastrointestinal tumour markers: CEA:23 ng/mL, CEA+. MUC6−	‐ Thymectomy+resection of adjacent mediastinal pleural, pericardium and left brachiocephalic vein, upper left lung lobectomy. Clear resection margins noted. ‐ Adjuvant carboplatin/docetaxel CTx+RTx. ‐ Subsequent development of bone and multi‐organ metastases. ‐ Changed to weekly paclitaxel CTx however further PD. ‐ Detected left pleural and meningeal dissemination later in the patient's clinical course. ‐ Irinotecan then trialled with minimal effect. ‐ DOD at 24 months.	24 months (DOD)
Seki et al. [76]	49M	Left shoulder pain	Mucinous thymic adenocarcinoma, stage: IVb	NA	+	NA	−	+	−	NA	‐ Tumour resection+adjuvant RTx 60Gy+ADOC CTx. ‐ Alive at 11 months with aggressive pleural metastases.	11 months (AWD)
Sawai et al. [77]	34M	New mediastinal mass	Tubular adenocarcinoma of the thymus, stage: IVa	NA	+	NA	+	−	NA	Gastroscopy+colonoscopy, CT: No potential gastrointestinal primary found Gastrointestinal tumour markers: CEA: 9.8 ng/mL, CEA+, CA19‐9: 42.3 U/mL, AFP WNL	‐ Tumour resection+resection of Superior vena cava and pericardium (due to involvement of disease). ‐ Adjuvant RTx+carboplatin/adriamycin/cyclophosphamide/vincristine CTx for 3 cycles. ‐ Pulmonary metastases developed at 8 months post‐operatively.	8 months (AWD)
Kapur et al. [78]	41M	NA	Mucinous thymic adenocarcinoma, stage: III	NA	Diffuse+	NA	Focal+	Focal+	NA	Endoscopy, radiology: No potential gastrointestinal primary found Gastrointestinal tumour markers: NA	‐ Neoadjuvant RTx due to large tumour bulk. ‐ Tumour resection 2 months post neoadjuvant RTx. ‐ Macroscopically, noted tumour extension into the inked margin along mediastinal vasculature. ‐ Subsequently received Adjuvant CTx+RTx. ‐ Developed a 1.5 cm right upper lung lobe metastasis after being symptom‐free for 19 months, to which the patient received a wedge resection. ‐ 1 year later, a second lung nodule was discovered in the right lower lobe. This nodule was resected, and the patient was given adjuvant RTx. ‐ Recurrence ‐free at 1 year after last surgery.	3 years+9 months (ACR)
Choi et al. [6]	15M	Dry cough	Mucinous thymic adenocarcinoma, stage: NA	NA	Focal+	NA	Focal+	Focal+	NA	Radiology: No potential gastrointestinal primary found Gastrointestinal tumour markers: NA	‐ Tumour resection. ‐ Residual anterior mediastinal mass noted on CT post resection. Therefore, received adjuvant RTx 64Gy. ‐ Developed bone metastases at 18 months. Received palliative RTx. ‐ DOD at 26 months.	26 months (DOD)

Abbreviations: +, positive; −, negative; A, alive; ACR, alive, in complete remission; ADOC, adriamycin+cisplatin+vincristine+cyclophosphamide chemotherapy; APR, alive, in partial remission; AWD, alive with disease; CTx, chemotherapy; CXR, Chest x‐ray; DOD, died of disease; F, female; FOLFIRI, irinotecan+leucovorin+5‐fluorouracil chemotherapy; FOLFOX, oxaliplatin+5‐fluorouracil+leucovorin chemotherapy; Fr, fractions; Gy, Gray; IHC, immunohistochemistry; IMRT, intensity‐modulated radiotherapy; M, male; NA, not available; NR, not relevant; PD, progression of disease; RTx, radiotherapy; SRS, stereotactic radiosurgery; WNL, within normal limits; XELOX, Oxaliplatin+Capecitabine chemotherapy.

Of note, Kalhor et al. [83] revisited 16 surgical pathology thymectomy cases from the corresponding author's centre from 2000 to 2016, whereby immunohistochemistry was performed in nine cases with a report of some cases staining positive for CDX2 and/or CK20. The exact number of cases staining for these markers is not documented, nor were the immunohistochemical features correlated with clinical notes, and thus this report was not included in Table 1 nor in the case count. Of the 49 cases described, one case was part of a report by Weissferdt et al. [82], who described 65 cases of thymic carcinoma that were studied with respect to their clinicopathologic and IHC characteristics. All tumors were surgically resected, with 1 mucinous adenocarcinoma showing positive staining for CDX2. The treatment and survival data were presented as a collective for the 65 cases, and therefore, the single case with a positive CDX2 expression could not be delineated with respect to the corresponding patient characteristics, treatment and survival outcome. Of the other 48 cases of enteric‐type thymic adenocarcinoma available in the literature, the median age of diagnosis was 52 years, ranging between 15 and 79 years. Twenty one of the forty‐eight patients were female, and 27 were male. These demographics are comparable to thymic carcinoma as a collective, with reports of median age at diagnosis between 54 and 65.5 years old, and a greater prevalence of disease in male patients [29].

The Masaoka stage at diagnosis was reported or could be determined from 33 cases. At diagnosis, three cases were Masaoka stage I, 3 cases stage II, 7 cases stage III, 3 cases stage IVa, and 17 cases stage IVb. CD5 status was reported in 37 cases. Twenty‐two cases showed any positivity for CD5, with 15 cases returning a CD5 negative status. While CK7 is classically negative in carcinomas of the thymus, [37] 19 of 40 cases showed any positivity for CK7, 21 cases were CK7 negative, and 9 cases did not report a CK7 status. TTF‐1 status was reported in 36 cases. Twenty nine out of 36 cases were negative for TTF‐1, and this is consistent with the current understanding that TTF‐1 is rarely expressed in thymic carcinomas [84, 85, 86]. Of note, it is not uncommon to observe TTF‐1 expression in thymomas [84, 87].

Of the 44 patients with documented treatment (Table 1), 29 received a tumour resection/thymectomy as part of their treatment. Among the 44 patients, 12 out of 14 with Masaoka stages I to IVa at diagnosis received a tumour resection/thymectomy, and 4 out of 15 diagnosed with stage Masaoka IVb disease also had a tumour resection/thymectomy as a part of their treatment [59, 70, 75, 76]. Two of these patients were alive with aggravated metastases at 11 months, [70, 76] one patient died from disease at 6 months postoperatively, [59] and one died from disease at 24 months [75]. Eleven other patients with stage IVb disease had their treatment regimen documented, whereby all 11 patients were treated with chemotherapy and/or radiotherapy without surgical resection.

In three reported cases, 5‐FU‐based chemotherapy was incorporated into the treatment regimen [21, 57, 68]. Gao et al. described the case of a 38‐year‐old man with metastatic enteric‐type thymic adenocarcinoma measuring 7.0 × 4.6 cm, with multiple muscle and bone metastases [21]. He was treated initially with first‐line carboplatin/docetaxel for 6 cycles, which did not result in a significant reduction in the mediastinal mass size on follow‐up CT. Subsequent mediastinal radiotherapy resulted in a slight reduction in tumour size. 9 months after presentation, a repeat PET‐CT demonstrated a new cerebellar metastasis, and progression of a right psoas major muscle metastasis. The patient underwent radiotherapy for the brain, psoas major, and lumbar vertebra metastasis, followed by 3 cycles of second‐line Nab‐paclitaxel/cisplatin+pembrolizumab. This regimen, however, did not provide effective control of the metastatic disease. The patient subsequently underwent FOLFOX+cetuximab for 3 cycles, resulting in a significant reduction of muscle metastases and pain relief. First‐line carboplatin/paclitaxel was used according to the NCCN guidelines for thymic carcinoma; however, their rationale for selecting second‐line nab‐paclitaxel/cisplatin+pembrolizumab was not provided. FOLFOX was selected in the third‐line setting due to the histological and immunohistochemical similarity between enteric‐type thymic adenocarcinoma and gastrointestinal adenocarcinoma. Cetuximab, an EGFR inhibitor, was added to the third‐line regimen as next‐generation sequencing (NGS) identified an EGFR copy number amplification. Li et al. described a case of a 44‐year‐old woman with an enteric‐type thymic adenocarcinoma measuring 43 × 38 mm, with metastases to the pericardium and sternum [57]. She was treated with 6 cycles of first‐line Capecitabine/Oxaliplatin (XELOX) with concurrent mediastinal radiotherapy (30Fr × 60Gray) during the first 3 cycles. Grade II myelosuppression and grade II radioactive esophagitis were experienced during chemoradiation. Intermittent tachycardia was also noted during the chemoradiation, to which the irradiation field was reduced to limit cardiac toxicity. A reduction in the size of the thymic mass to 37 × 22 m was observed following the 6 cycles of XELOX, and her disease remained stable at 16 months of follow‐up. XELOX, comprising capecitabine and oxaliplatin—with capecitabine serving as an oral prodrug of 5‐FU [88]—was selected due to the histologic resemblance between enteric‐type thymic adenocarcinoma and colorectal adenocarcinoma. Sawaki et al. reported the case of a 59‐year‐old man presenting with a 76 × 63 × 31 mm enteric‐type thymic adenocarcinoma with multiple bone metastases, and associated pericardial and right pleural effusion [68]. He was treated with first‐line XELOX, with radiotherapy to a vertebral metastasis during Cycles 1 and 2 to alleviate back pain. Following Cycle 2, there was a temporary decrease in the patient's right pleural effusion and serum biochemical markers, however, these worsened prior to Cycle 3. The cancer pain worsened after Cycle 4 of XELOX, in addition to a worsening liver function derangement, which the treating team clinically assessed as disease progression, although no imaging was reported to have been performed at the time. Second‐line FOLFIRI+cetuximab was commenced, whereby the LDH levels normalised and ALP levels significantly reduced. The treatment was complicated by an interstitial pneumonia and a suspected Pneumocystis jiroveci pneumonia following the second cycle of FOLFIRI+cetuximab. Following antibiotic and steroid treatment, he resumed treatment with FOLFIRI alone, before ceasing treatment altogether due to a worsening functional status, dying of disease at 6 months after initial diagnosis. An autopsy reported metastases to the lung, liver, bone, right adrenal gland and bladder. As there was no established treatment protocol for thymic adenocarcinoma with enteric features, the authors decided on XELOX and FOLFIRI in the first and second‐line settings, respectively. Their decision was guided by an article by Varadhachary et al., which suggested that carcinomas of unknown primary with a colon cancer–like immunophenotype‐ specifically CDX2+/CK20+/CK7−, may benefit from chemotherapy regimens typically used for colorectal cancer [89]. This immunohistochemical profile matched that of the patient described by Sawaki et al. Cetuximab was added to FOLFIRI by Sawaki et al. as their patient's disease was KRAS wild‐type, whereby Cetuximab is an accepted additive to 5‐fluoropyrimidine‐based chemotherapy in pMMR, KRAS wild‐type, unresectable or metastatic colon cancer [22]. It is, however, difficult to extrapolate survival outcomes from the available data on 5‐FU‐based chemotherapy in enteric‐type thymic adenocarcinoma, due to the small sample size treated with this chemotherapy. The three aforementioned cases treated with 5‐FU‐based chemotherapy were stage IVb at diagnosis. Although we could not confer a definite stage to our patient at initial diagnosis in May 2024, she did not demonstrate radiological evidence of distant metastasis at diagnosis, and her pericardial effusion cytology in October 2024 was negative, suggesting that did not yet have stage IV disease at diagnosis, and therefore, we cannot parallel our patient with these three cases.

Common side effects across all chemotherapies include fatigue, mucositis, gastrointestinal disturbances, myelosuppression, and alopecia [90]. Fluoropyrimidines can confer grade 3 or 4 toxicities‐ primarily myelosuppression or gastrointestinal side effects in 10%–30% of patients [91]. Our patient experienced myelosuppression, whereby her neutrophil and platelet count began to decline prior to her third cycle of FOLFOX. Her lowest recorded neutrophil count was 0.41 × 10^9^/L (RR: 2.0–8.0 × 10^9^/L) in early August 2024, coinciding with her hospital admission for a febrile illness later confirmed as a staphylococcal bacteraemia. The febrile neutropenia and her prolonged recovery from the bacteraemia led to a deferral of her fourth cycle of FOLFOX. Of note, dihydropyrimidine dehydrogenase (DPD), encoded by the DPYD gene, is the rate‐limiting enzyme for fluorouracil metabolism, and catabolises approximately 85% of fluorouracil. 3%–6% of the general population have defective variants of the DPYD gene and are at risk of significant fluoropyrimidine toxicity [92, 93]. Genotyping for these variants may assist in treatment selection between fluoropyrimidine‐based regimens and alternatives such as carboplatin/paclitaxel+ramucirumab. Oxaliplatin, a component of FOLFOX, has a dose‐limiting neurotoxicity, [94] typically manifesting as sensory dysfunction, cold dysesthesias, cramps, or muscle spasms. These are often acute and reversible in the earliest courses of treatment; however, as with all platinum agents, there may be a gradual progression of symptoms for weeks to months after drug cessation [95]. Carboplatin is less often associated with neurotoxicity than oxaliplatin, [94, 95] and its dose‐limiting toxicity is myelosuppression. Paclitaxel, however, is strongly associated with peripheral sensory neuropathy, which is often dose‐related, and less common at lower doses of 135–200 mg/m^2^ [96]. Therefore, both the FOLFOX and carboplatin/paclitaxel+ramucirumab regimens carry risks for neuropathy. A secondary alternative to carboplatin/paclitaxel+ramucirumab listed by NCCN for the upfront treatment of unresectable thymic carcinoma is that of etoposide/cisplatin, [7] however, cisplatin itself also has a high neurotoxic profile [95]. When commencing on any of these regimens, there should be close monitoring for neurotoxic symptoms, and prompt consideration of dose reduction or discontinuation if neuropathy emerges. Alternatively, fluorouracil/leucovorin/irinotecan (FOLFIRI), another standard systemic regimen used in colonic adenocarcinoma [22] may be a viable alternative, as it excludes oxaliplatin and thus may pose a lower risk of neurotoxicity. While our patient responded to FOLFOX, she decided to cease chemotherapy after cycle 4. This was driven by her functional decline from fatigue and reduced exercise tolerance due to dyspnoea, which was attributed to multiple factors, including her underlying cancer, SVC syndrome, non‐specific side effects of chemotherapy, her prolonged admission for bacteraemia, and recurrent pericardial effusions. She had also developed a mucositis and a neuropathy after cycle 3, which later self‐resolved.

Among the preferred second‐line systemic options listed by the NCCN for thymic carcinoma, [7] gemcitabine±capecitabine, or pembrolizumab monotherapy also have established roles in some gastrointestinal adenocarcinomas. These agents, as such, may be reasonable second‐line therapy options for patients whose tumors exhibit an enteric‐type immunohistochemistry, but for whom the histopathology, other immunoprofiling, and gastrointestinal workup are inconclusive in determining whether the primary site is thymic or gastrointestinal. Gemcitabine‐based chemotherapy is a recommended treatment in pancreatic adenocarcinoma when metastatic activity is discovered following surgical resection, and within 6 months of primary therapy completion [23]. Gemcitabine/capecitabine combination chemotherapy is one of the preferred regimens in the adjuvant treatment of biliary tract cancers [24]. Pembrolizumab monotherapy has utility in dMMR or high mutational burden tumors across several gastrointestinal adenocarcinomas, including gastric, [25] colonic, [22] pancreatic, [23] and biliary tract cancers, [24] whereby the high somatic mutations in dMMR disease result in a higher neoantigen load that is more amenable to immunotherapy [97]. Pembrolizumab may thus be a reasonable second‐line option if the tumour in question is demonstrated to be dMMR or has a high tumour mutational burden.

Platinum/taxane chemotherapy was incorporated into the treatment regimen in 12 cases [20, 21, 54, 56, 60, 64, 67, 74, 75]. Partial response to paclitaxel/carboplatin was reported in three cases [54, 56, 64], with 9 cases noting disease progression or a lack of clinical effect whilst on or after platinum/taxane chemotherapy [20, 21, 60, 67, 74, 75]. Ersoz et al. reported a patient with stage III disease who underwent a tumour resection with adjuvant radiotherapy, with subsequent development of two brain metastases 6 months post‐operatively. The patient then underwent chemoradiation with four cycles of carboplatin/paclitaxel, with complete resolution of the two metastases but development of two new brain metastases at 12 months post tumour resection [56].

The NCCN guidelines have previously advised carboplatin/paclitaxel as the first‐line systemic therapy of choice in unresectable or metastatic thymic carcinomas, and have recently added ramucirumab to this regimen [7]. Vascular Endothelial Growth Factor (VEGF) has been reported to be overexpressed in thymic carcinomas, [98] and there has been increasing interest in anti‐angiogenic agents in the treatment of thymic carcinomas. Phase II trials have demonstrated the efficacy of sunitinib [99] and Lenvatinib [100] in the previously platinum‐treated thymic carcinoma and unresectable or metastatic thymic carcinoma, respectively. Ramucirumab, a VEGF‐Receptor 2 antibody, was added to carboplatin/paclitaxel regimen in the NCCN guidelines for the upfront treatment of unresectable or metastatic thymic carcinoma, based on its promising activity in the RELEVANT phase II trial. This combination demonstrated an overall response rate of 80.0% (*p* > 0.0001 vs. a null hypothesis ORR of 20%) in previously untreated metastatic thymic carcinoma [101]. Paclitaxel itself also has antiangiogenic properties, and there may be a synergistic effect when combining paclitaxel and other antiangiogenic therapies [101]. There have also been other agents studied in phase II trials for the treatment of thymic carcinoma. While not yet listed as a standard option, carboplatin/paclitaxel+atezolizumab, an anti‐PD‐L1 antibody, showed promise in the recent MARBLE trial, demonstrating a higher objective response rate of 56% (Fisher's exact test *p* < 0.0001) relative to historical controls assessing the use of carboplatin/paclitaxel alone. A post hoc analysis examining PD‐L1 expression did not identify a statistically significant difference in median PFS across patients stratified by PD‐L1 expression levels (< 1%, ≥ 1% to < 5%, ≥ 5% to < 50%, or ≥ 50%) [13]. However, in a phase II trial by Giaccone et al. in patients who had progressed on first‐line chemotherapy, a higher partial or complete response was observed with pembrolizumab‐ an anti‐PD‐L1 receptor antibody in patients with high (> 50%) PD‐L1 expression compared to those with low (1%–49%) or no PD‐L1 expression [102]. Accordingly, it may be beneficial to offer anti‐PD‐L1 immunotherapy in patients with a high tumour PD‐L1 expression, particularly after disease progression on first‐line chemotherapy. The CAVEATT phase II trial assessed the combination of the anti‐PD‐L1 antibody avelumab with the VEGF‐receptor inhibitor axitinib in thymic carcinoma patients who had disease progression after at least one line of platinum‐chemotherapy, demonstrating an overall response rate of 34% [103]. This regimen is now listed as a secondary option in second‐line therapy for unresectable or metastatic thymic carcinomas under the NCCN guidelines [7]. It should be noted that the aforementioned trials incorporating immunotherapy or antiangiogenic therapy alongside carboplatin/paclitaxel were performed on patients with thymic carcinomas, without stratification of carcinomas subtype, and their effect on thymic adenocarcinomas and specifically, enteric‐type thymic adenocarcinomas, is not reported. While the three previous reports describing fluoropyrimidine‐based chemotherapy [21, 57, 68] and our current report have all described some response in their use in enteric‐type thymic adenocarcinoma, the limited number of cases makes it difficult to compare the response and survival outcomes between this regimen and that of carboplatin/paclitaxel in enteric‐type thymic adenocarcinoma. Furthermore, with the recent addition of ramucirumab to the carboplatin/paclitaxel regimen in the NCCN guidelines for the treatment of unresectable or metastatic thymic carcinoma, future research evaluating the efficacy of fluoropyrimidine‐based chemotherapy should ideally use this updated combination as the comparator. Ultimately, it would be beneficial for future research to compare the survival outcomes of carboplatin/paclitaxel+ramucirumab with those of standard gastrointestinal carcinoma regimens, such as FOLFOX, FOLFIRINOX, or FLOT in the treatment of primary enteric‐type thymic adenocarcinoma.

## 4 Conclusion

We describe the 50th reported case of primary enteric‐type thymic adenocarcinoma, and the first involving unresectable disease treated with first‐line FOLFOX chemotherapy. Our patient maintained a response to treatment despite a prolonged treatment interruption and continued to maintain disease stability for 6 months after discontinuing treatment. While enteric‐type thymic adenocarcinoma is rare, previously reported systemic treatments have predominantly utilized carboplatin/paclitaxel, which until recently, was the regimen recommended by the NCCN guidelines for thymic carcinomas. This recommendation has recently been succeeded by carboplatin/paclitaxel+ramucirumab [7]. It should be noted, however, that prospective trials studying carboplatin/paclitaxel in thymic carcinoma treatment are largely based on the squamous subtype [11] or undefined subtypes [10] and thus their applicability to thymic adenocarcinomas, particularly the enteric‐type, is unknown. Three prior studies have reported on the use of 5‐FU‐based regimens in enteric‐type thymic adenocarcinoma, each also inducing at least a partial response to suggest the potential activity of 5‐FU regimens in this malignancy [21, 57, 68]. While the case number remains too small to draw conclusive evidence regarding the superiority of FOLFOX over carboplatin/paclitaxel, our report of a patient whose disease responded whilst on FOLFOX chemotherapy suggests that 5‐FU‐based regimens may be a viable alternative and may be worth considering as a first‐line option or second‐line option following disease progression on carboplatin/paclitaxel+ramucirumab. It should be noted, however, that it is unclear how much of our patient's disease stability could be attributed to her FOLFOX chemotherapy, palliative radiation therapy, indolent disease, or a combination of these factors. It is also unknown how much longer she might have maintained a treatment response or stable disease had she continued FOLFOX beyond cycle 4. The differences between 5‐FU uptake in the thymus and the gastrointestinal epithelia are not well established, and we encourage further reporting of cases of enteric‐type thymic adenocarcinoma treated with 5‐FU‐based regimens such as FOLFOX, in any line of therapy, to better inform its efficacy in this rare malignancy.

## Author Contributions

**Carl He:** conceptualisation, writing – original draft, review and editing, investigation, methodology. **Georgia Bentick:** conceptualisation, supervision, writing – review and editing, investigation, methodology. **Patrick Hosking:** conceptualisation, writing – original draft, review and editing, investigation. **Andrew Mant:** conceptualisation, writing – review and editing, supervision, investigation.

## Consent

Written consent was obtained from the patient prior to the submission of this manuscript.

## Conflicts of Interest

The authors declare no conflicts of interest.

## Data Availability

Data sharing not applicable to this article as no datasets were generated or analysed during the current study.
